# The Future Hospital in Global Health Systems: The Future Hospital Within the Healthcare System

**DOI:** 10.1002/hpm.3891

**Published:** 2025-01-15

**Authors:** N. J. Sebire, A. Adams, L. Celi, A. Charlesworth, M. Gorgens, M. Gorsky, O. Landeg, Y. Nagasawa, K. T. Nimako, C. Onoka, S. Roder‐DeWan, N. Watts, M. McKee

**Affiliations:** ^1^ Digital Research and Informatics Unit NIHR Great Ormond Street Biomedical Research Centre at UCL London UK; ^2^ Department of Social Studies of Medicine / School of Architecture McGill University Montreal Canada; ^3^ Laboratory for Computational Physiology Massachusetts Institute of Technology Cambridge Massachusetts USA; ^4^ Harvard T.H. Chan School of Public Health Boston Massachusetts USA; ^5^ REAL Centre Team The Health Foundation London UK; ^6^ Digital Health Programme World Bank Group Washington DC USA; ^7^ Centre for History in Public Health London School of Hygiene and Tropical Medicine London UK; ^8^ Faculty of Public Health and Policy London School of Hygiene and Tropical Medicine London UK; ^9^ Department of Architecture University of Tokyo Bunkyo City Japan; ^10^ Health Nutrition and Population Global Practice The World Bank Washington DC USA; ^11^ College of Medicine University of Nigeria Nsukka Nigeria; ^12^ Global Practice Service Delivery, Health, Nutrition and Population The World Bank Washington DC USA; ^13^ NHS England London UK; ^14^ Centre for Sustainable Medicine National University of Singapore Singapore Singapore; ^15^ Department of Health Services Research and Policy London School of Hygiene and Tropical Medicine London UK

**Keywords:** financing, future hospital, healthcare system, planning, sustainability

## Abstract

Future hospitals must be able to adapt in many ways to the changing demands on their roles and functions within evolving healthcare delivery infrastructures. These include changing population structures and needs, new models of healthcare provision, technological advances, and innovations in design, all while enhancing their environmental sustainability. This article sets out the issues that those determining healthcare policy and designing future hospitals must consider if they are to become and remain fit for purpose within the wider health and social care system. It also examines the need for, and challenges to, strategic healthcare planning, creating future hospitals that are sustainable, net‐zero carbon organisations, and ensuring resilience in the face of a range of potential shocks. Future hospitals play a crucial role in healthcare worldwide, regardless of the country's income level. Hospitals cannot be viewed without broader health system changes, infrastructure, community and cultural factors, staffing and other considerations. We anticipate that future hospitals will enhance population health in all settings and support the move towards more consumer‐centric healthcare. We urge clinical and policy planners to consider the factors discussed carefully to maximise the benefits.

**Summary**

- Planning the hospital system of the future must take a systems approach, making use of the increasingly sophisticated models of healthcare demand, while incorporating sufficient flexibility for a range of possible, but unpredictable scenarios.
- Future hospital planning requirements will vary significantly among and within HICs and LMICs, reflecting a wide range of sociodemographic factors
- Multiple hospital financing approaches are possible, each with near and long term consequences
- Future healthcare organisations must not only promote health and well‐being but also protect their communities through thoughtful building and organisational design
- Future hospital function requires sufficient trained healthcare staff, digital literacy for both staff and patients, and adequate education and training

## 1 Introduction

Future hospitals must adapt to developments in population needs, care models, technology, design, and planning rules [1]. This multidisciplinary perspective explores how hospitals can address these challenges while emphasising their role within broader health and social care systems. Key considerations include external factors, like achieving sustainability and net‐zero carbon status, and internal factors, such as financial planning, staffing, and human resources. The article synthesises insights from researchers across disciplines to provide a practical foundation for discussions on the hospital of the future.

Factors such as climate change, sustainability [2], technological advancements [3], and lessons from COVID‐19 [4, 5] highlight the urgency of rethinking hospital planning and operations. This applies to both high‐ and low‐income countries facing challenges in integrating hospitals into broader health systems. Failing to leverage opportunities risks compromising future health systems' capacity to meet demand and respond to emergencies.

## 2 Methods

Our approach is described in the accompanying paper. In brief, as a future‐looking perspective that requires thinking outside the box, our approach is informed by certain principles. Prediction is speculative, hospitals are part of complex systems, and changes are non‐linear and the consequence of multiple interacting factors. Hence, we selected a panel of authors with expertise in clinical medicine, architectural and public health history, architecture and wellbeing and urban design, sustainability, data science and artificial intelligence, economics, and health systems. Each contributed insights into likely futures for the hospital that were then integrated into a narrative text.

## 3 Future Hospital Planning

Healthcare planning seeks to deliver quality care to the right people in the right place, efficiently using available resources. Hospital design has historically remained static, with at most incremental changes or crisis responses, lacking long‐term strategies and producing outdated designs with wards, labs, and surgical theatres operating independently. However, innovative designs like the Friendship Hospital in Bangladesh, recognised by the Royal Institute of British Architects (RIBA) in 2021, which incorporates a simple, functional plan and cost‐effective solutions to issues like ventilation, providing an uplifting atmosphere for the local community, demonstrate cost‐effective, functional, and uplifting approaches for rural populations [6]. Future planning must address hospitals' roles in diverse health systems, adapting to demographic shifts and evolving care needs.

The COVID‐19 pandemic underscored hospitals' critical role while exposing system limitations like unequal access [7]. Both the World Health Organisation (WHO) [8] and Healthcare Information and Management Systems Society (HIMSS) [9] advocate for strategic planning using data, digital technologies, and workforce innovations to reshape care delivery. Hospital capacity must align with population demand, considering sociodemographic factors, disease burden, and alternatives like community care. Hospital capacity should align with population demand, factoring in sociodemographic and disease burdens. Despite ageing populations, many HICs have reduced hospital beds per 1000 population—from 9.6 in the 1960s to 4.2 in 2021, varying from 12.6 (Japan) to 2.4 (UK) [10]. Conversely, some LMICs, like Nigeria, have increased bed numbers by 4% annually to meet WHO targets [11]. However, bed counts are a poor indicator of hospital capacity [12], as highlighted during the pandemic when rapidly constructed facilities lacked the necessary staff and functions like labs and operating theatres. Needs also depend on population characteristics and alternatives like community or social care. Additionally, beds are ineffective if patients cannot afford care, a common issue in LMICs with out‐of‐pocket payments.

Reduced bed capacity can be beneficial, reflecting decreased disease burden, alternative care options, or digital technologies enabling at‐home care. For example, Estonia halved its hospitals over a decade while enhancing community care and technology, achieving one of the EU's lowest unnecessary admission rates [13]. However, changes should consider the risks of creating a ‘digital divide’, as some groups lack access to smartphones or data [14]. Preventative and community care holds significant potential to lower inpatient demand. Those remaining in hospitals often need highly complex care as admissions and lengths of stay decline.

Future hospital systems must integrate advanced systems to anticipate and adapt to changing demand, similar to how the retail sector uses meteorological and other data to anticipate demand for certain products [15]. Seasonal demand spikes, such as in winter, are complicated by the post‐pandemic lack of COVID‐19 seasonality compared to influenza. Over time, home‐based care, patient preferences, and choices will reshape demand. The pandemic also exposed many countries' inadequate surge capacity. However, prediction is imperfect, and planners face constraints like location, space, budgets, and inflexible facilities, highlighting the need for future‐proof designs [16].

Balancing design constraints with accessibility is challenging. Building on greenfield sites is cheaper but risks excluding those without transport, especially in LMICs [17]. Urban hospitals offer economies of scale but disadvantage rural populations [18], while inadequate public transport and sustainable options undermine accessibility and sustainability goals. Disparities also affect HICs, where Indigenous peoples face barriers due to complex care needs and the difficulties of travel from remote locations [19]. Cultural and linguistic diversity exacerbate access issues; for instance, in Australia, limited English proficiency correlates with higher rates of chronic conditions [20].

To support universal health coverage, future hospitals must integrate into evolving healthcare systems, including social, public health, primary care, varied care models, technology, and consumer preferences. Balancing these elements is vital since inadequate primary care increases hospital demand, while hospital projects often lack system‐wide impact planning due to organisational constraints [21]. Hospitals differ in dimensions like organisational structure (independent or networked), estates (single or multiple sites), technology connectivity, funding models, and scope of practice, all of which influence planning. In rural LMICs, infrastructure like electricity, broadband, and water shapes hospital location and design, benefiting local communities and healthcare quality [22].

A UK review advised against over‐optimistic assumptions about future demand, noting past plans overlooked new diseases, treatments, technology, and staffing needs. These oversights led to delays, budget overruns, and insufficient multidisciplinary expertise [23].

## 4 Capacity Planning for Future Hospitals

Hospitals are an important contributor to total healthcare costs, accounting for around one‐third of overall costs across HICs and LMICs [24, 25]. This is broadly reflected in hospital beds per capita. Still, there is only a weak association between health spending and hospital bed numbers since there has been a reduction in the average hospital length of stay and more treatment provided in ambulatory settings. Patients are also increasingly present with complex conditions requiring extended, high‐intensity hospital care but with less recuperating time. This results in marked variations in hospital capacities; Denmark, Sweden and the Netherlands have below‐OECD average numbers of hospital beds but above‐average health spending by providing relatively more care in community and ambulatory settings [24].

## 5 Hospital Markets

The hospital sector has been consolidating worldwide alongside reduced capacity. Evidence on optimal hospital size suggests economies of scale up to 200 beds and diseconomies beyond 600 beds [26]. Between 2010 and 2017, nearly 800 hospital mergers occurred in the USA, reducing competition. Single systems now account for over 50% of inpatient admissions in most markets [27]. Denmark restructured its hospitals to enhance access and quality by merging facilities, closing smaller ones, and centralising specialised services, but the changes yielded mixed outcomes [28].

Restructuring often expands hospital size and integrates facilities into networks. In France, Germany, and the USA, most hospitals now belong to systems or partnerships, while UK hospitals increasingly form networks, though evidence of improved outcomes is lacking [29]. The UK NHS shows restructuring is not simply a technocratic process without consequences [30], as communities often resist hospital rationalisation plans [31], emphasising the need for local input in decision‐making. New Zealand's centrally funded hospital system also balances local and national control, highlighting tensions between grassroots democracy and centralised planning [32].

Several factors drive hospital mergers and network growth. In France, financial pressures led to small private hospital closures, while in Germany, reduced public funding and patient competition amid excess capacity were vital. In the US, economies of scale, market forces, negotiating power, and the Affordable Care Act spurred mergers. The US Medicare Payment Advisory Commission concluded that the ‘preponderance of evidence suggests that hospital consolidation leads to higher prices’ [33]. Regulatory efforts to improve quality and safety also drive consolidation in France, Denmark, and Germany [34]. These trends extend to LMICs, where financial motives influenced mergers in Nigeria, and market concentration grew in South Africa [35, 36].

Applying new public management models to healthcare systems has also affected how hospitals are organised and managed [37], with public hospitals in some countries adopting quasi‐market mechanisms for investment and funding. Examples include UK Foundation Trusts and independent public hospitals in Germany. For‐profit hospitals have expanded across Europe, now representing significant shares of hospital beds (25% in France, 33% in Germany) [34]. In Germany, two privatisation waves, post‐reunification in the 1990s and later financial crises saw private chains acquire struggling hospitals and consolidate the private sector into large groups [38].

## 6 Geographic and Political Influences

Future hospital requirements vary across HICs and LMICs, shaped by diverse factors. LMICs often face a dual disease burden: ageing populations with non‐communicable diseases alongside infectious diseases and poor maternal and child health [39]. While primary healthcare is critical for universal coverage, advanced care, emergencies, and complex disease management require robust generalist and specialist hospital services [40, 41], yet up to 90% of people in LMICs lack adequate surgical care access [42, 43, 44].

Geographic disparities worsen access issues. WHO recommends one hospital providing comprehensive obstetric and neonatal care per 500,000 people [45], often met nationally in LMICs but not regionally. Two‐thirds of global adverse events occur in LMICs, where critical conditions often go untreated due to workforce shortages and inequitable specialist distribution. For instance, patients with acute myocardial infarction may not receive reperfusion therapy [46, 47], and 90% of critically ill patients with low oxygen levels or low blood pressure do not receive appropriate treatment [48]. These challenges are aggravated by skilled healthcare staff migrating to HICs to address their shortages [49, 50].

However, the debate over centralising hospital provision is contentious, with arguments for and against it. Supporters of centralisation cite several benefits. Centralised hospitals tend to achieve better clinical outcomes due to higher procedure volumes, which enhance patient safety. For example, hospitals performing more complex surgeries, like coronary artery bypass grafting, see improved success rates [51]. Additionally, centralisation allows for cost savings by consolidating resources like medical staff and equipment, reducing overall healthcare expenses. Specialised expertise is another advantage, as centralised facilities can develop advanced knowledge and protocols, improving patient care and reducing mortality.

Critics, however, highlight significant downsides. Concentrating hospitals in urban areas risks creating rural ‘deserts’, with longer travel times and poorer quality of care [45, 52]. This ‘distance decay’ effect can result in the underutilisation of healthcare by those living far from these centres and the underutilisation of healthcare by those living far from these centres [53]. Centralisation may also lead to higher staff turnover, as increased workload and stress can impact healthcare workers' job satisfaction and care quality. Furthermore, some argue that over‐centralisation may not always improve outcomes, especially in well‐organised healthcare systems [54]. Thus, while centralisation offers clear benefits in outcomes and cost‐efficiency, the potential drawbacks, such as accessibility issues and workforce challenges, must be carefully considered, along with innovative thinking about opportunities stemming from advances in digital technology, such as those using telemedicine.

Finally, the COVID‐19 pandemic highlighted the need for pandemic preparedness everywhere [5] but especially in hospitals in LMICs, emphasising inclusive systems with infection control, resilient supply chains, capacity, and regulation to manage outbreaks effectively [55].

## 7 Hospital Funding

Hospitals require reimbursement to cover costs, including initial capital and sustained investments [56]. Payment rates must ensure quality, efficient, and patient‐responsive care. Unlike competitive markets, hospitals often rely on third‐party payers, including private insurers, social insurance, or tax‐funded bodies. Payment systems have evolved from fee‐for‐service and global budgets to activity‐based models using case‐mix measures like diagnosis‐related groups (DRGs) [57], which promotes transparency and incentivises efficient quality care. Other models include pay‐for‐performance, continuum‐of‐care funding, and capitated budgets [58], but challenges remain with balancing rewards, penalties, and outcome attribution [59].

Future hospital programs are major infrastructure projects in HICs and LMICs, costing €200–€800 million (2023 costs) in HICs [60] and about a 10th in LMICs [61]. Four essential functions—finance, design, building, and operations—are handled by different organisations based on ownership and capital‐raising methods. Some countries have attempted to integrate these functions, creating consortia of banks, architects, construction and facilities management companies as special‐purpose vehicles [62]. In others, the hospital organisation may retain some functions, such as issuing equity or bringing operations in‐house.

Raising capital can be challenging, as the Private Finance Initiative (PFI) used in the UK, Portugal, and elsewhere shows [63]. PFI schemes shifted upfront costs to private funding, repaid over years with interest. The model was attractive in the circumstances prevailing in the UK in the 1990s, as costs were moved from the government's balance sheet, impacting apparent national debt calculations [64]. The benefit was building hospitals that could not otherwise be funded by shifting costs to future years. Although the UK stopped using the PFI scheme in 2018, and Australian schemes have been bought out, many British hospitals retain PFI contracts with associated debt, paying for increasingly obsolete facilities [65]. Income generation for hospital programs typically combines public and private sources. Some HICs are adopting payment systems covering entire care pathways, though these pose challenges when benefits span multiple system areas [66].

## 8 Future Hospital Sustainability

Climate change significantly impacts patients, public health, and health systems, with two‐thirds of infectious diseases expected to intensify due to climate change [67]. Sustainable healthcare aims to protect community health while minimising its environmental harm. Addressing climate change offers opportunities for public health intervention [2]. Future healthcare organisations must promote well‐being and safeguard communities through resilient building and organisational design [16]. Climate change affects physical and mental health via rising temperatures, extreme weather trauma, and livelihood losses, disproportionately impacting older adults, children, and those with pre‐existing conditions. Additionally, extreme weather events may disrupt healthcare delivery, necessitating climate‐resilient systems.

Health systems significantly impact the environment, accounting for 1%–5% of global climate impact, more for some nations [68]. The healthcare sector would be the fifth‐largest CO2 emitter if it were a country [69]. In the UK, estates account for 15% of NHS emissions, primarily from energy use [70]. Environmental health issues, such as pollution, contribute to conditions like heart disease, asthma, and cancer. Transitioning to a net‐zero economy can improve health through reduced pollution and increased physical activity, making healthcare systems pivotal in reducing environmental impacts.

The COP26 Health Programme introduced initiatives for climate‐resilient, sustainable health systems [71]. The UK's Greener NHS initiative aims for net zero by 2040, with significant carbon reductions by 2028–2032 [70]. By 2022, NHS efforts cut emissions equivalent to powering 1.1 million homes annually, reducing carbon emissions by 30% over a decade. Achieving sustainability in healthcare requires collaboration across regions and nations, considering diverse challenges in HICs and LMICs. These include sustainable hospital infrastructure, transportation, procurement, and healthcare models that reduce environmental impact [72], including the impact of digital and informatic systems [73], as well as integrating hospitals into sustainable urban infrastructures.

While there are many common aspects to creating sustainable hospitals, considering the facility's entire life cycle and embracing eco‐friendly construction, energy conservation, and sustainable waste management, there are also specificities related to geography and climate [74]. Thus, the construction methods employed in high latitudes will differ from those in the tropics [75]. However, this is complicated further by the realisation of the importance of ventilation in hospitals, re‐learnt during the COVID‐19 pandemic [76]. Consequently, while natural ventilation will reduce risks of infection, it will not always be possible, and alternative methods are required.

Decarbonising hospital estates is cost‐effective and improves energy efficiency, air quality, and comfort. The UK NHS net‐zero Building Standard focuses on reducing operational energy use, embodied construction carbon, and whole‐life carbon for net‐zero buildings. These standards apply to NHS buildings and refurbishments, advancing a whole‐life carbon approach.

## 9 Health System Resilience

Health system resilience refers to the ability of health institutions and populations to respond to crises while maintaining core functions [5]. Future hospitals must withstand climate‐related hazards and pandemics without compromising care or patient safety, even during simultaneous events. This requires systems and infrastructure to support response and recovery, aiming for “turbulence‐ready” health systems capable of handling future disruptions [77], acknowledging health systems' complexity and interdependencies.

Hospitals must prepare for climate change‐related weather events like flooding and heat waves, staying resilient to current threats while addressing emerging challenges. Hospital design should allow flexibility for surge capacity, such as repurposing facilities when needed. Healthcare must take a long‐term, strategic approach to ensure mitigation efforts avoid unintended consequences, inequalities, and policy lock‐in.

The healthcare workforce remains crucial for future hospitals' resilience. Hospitals worldwide will face increasing workforce challenges, including staff shortages, an ageing workforce, and the need for ongoing professional development in new technologies and care models. Thoughtful facility design and supportive organisational cultures can help reduce burnout and retain staff [78]. LMICs are especially affected by skilled staff shortages, particularly in rural areas, due to limited access to services like education for their families and safety and security concerns [79]. The lack of younger healthcare workers may worsen with migration. Insufficient staffing leads to overburdened healthcare professionals, burnout, migration of trained staff, and reduced care quality [80].

## 10 The Global Context

In the early twentieth century, many hospitals operated independently rather than as parts of a coordinated health system. As health systems became more coordinated, governments played more prominent roles in regulating, funding, and providing healthcare. One early example was New Zealand's national health service in 1938 [81, 82], with the UK following later [83]. While arrangements vary, a growing share of health expenditure in most countries now comes from the government, even in those with social or insurance systems. The WHO has promoted national planning for estimating population needs and healthcare services, though resource limitations and political factors limited its impact. By the 1970s and 1980s, the ‘health system’ concept emerged, incorporating national or regional planning across medical inputs, processes, and outcomes.

Those who can afford healthcare typically need it least, making organised health systems a form of resource redistribution, which is inherently political. This is reflected in international commitments to universal health coverage. However, powerful individuals and groups, often through think tanks, media control, and political donations, argue otherwise. From the 1980s, there was a shift towards market‐based systems, often referred to as the Washington Consensus [84], based on the belief that efficiencies could be achieved by increasing private and non‐profit hospital services, with the state focused on market failures. This was especially prominent in Anglophone countries and was sometimes described as rowing rather than steering [85], or buying rather than making [86], Items like hospital furniture should be bought where clearly specified, minimising opportunities for exploitation [87]. The challenge is that medical care is difficult to specify and prone to opportunistic behaviour [88], such as manipulation of payment codes [89].

The rise of chronic infectious diseases like HIV/AIDS, malaria, and TB led to greater emphasis on vertical disease‐specific health programmes rather than health system development. This was particularly evident in the Millennium Development Goals (2000–2015), which focused on infectious diseases and maternal/child health. Subsequent Sustainable Development Goals (SDGs) (2015–2030) partially addressed this but still emphasised disease categories, with a limited explicit focus on health system organisations, especially regarding future hospital planning. While progress is being made, challenges persist due to global financial crises and the COVID‐19 pandemic. As mentioned, healthcare is political, and in some countries, powerful interests are trying to roll back coverage [90, 91] by creating divisions that label some groups, often migrants, as ‘undeserving’ while enabling those who can afford to pay to opt‐out [92].

Future hospital planning will vary significantly between countries due to differences in resources, systems, geography, and politics. In some African countries, about one‐third of the population is more than 2 hours away from the nearest hospital, with marked variation between nations [93]. Physical access to care is limited in many areas; smaller hospitals improve access to emergency care but often have limited capabilities, particularly outside large cities in Africa [94]. Reliable infrastructures, like clean water and stable electricity, are essential for hospitals to function. A review of electricity supply in LMICs' hospitals performing surgery found that only about 60% had a continuous supply, and 70% had generators. Continuous supply rates ranged from near 0% (Sierra Leone) to 100% (Iran) [95]. Improving such infrastructures is critical in some regions. Similarly, transport infrastructure is crucial to ensure patient and staff access and secure, predictable supply chains [96].

Just as LMICs leapfrogged landlines to mobile phones, digital technologies in these countries have particular benefits, including increased access to care, faster, more accurate case identification, screening, contact tracing, diagnosis, outbreak prediction, vaccine formulation, reduced workload for healthcare professionals, and support for preventive care [97, 98]. Telemedicine and digital health are increasingly used in LMICs through video consultations, online decision support, text messaging, information provision, and online therapy. However, implementing these technologies at scale faces challenges, such as poorly coordinated health systems, lack of quality data, inadequate infrastructure like electricity and internet connectivity, and limited interoperability [99]. Return on investment calculations raise questions about whether traditional funding models are suitable in LMICs and if new models are needed to achieve benefits at scale [100].

In LMICs, balancing future hospital development with limited funding is challenging. Public‐private partnerships can be attractive, facilitating the rapid creation of healthcare facilities. However, as in HICs, there are downsides, including reduced flexibility, financial sustainability concerns, and risks of investor‐state dispute procedures, where powerful global corporations may challenge governments. In Lesotho, a single privately financed hospital consumed half of the national health budget [101]. Future hospitals must be adaptable to changes in disease patterns, scientific advances, new care models, and financial contexts. These schemes can limit that adaptability, although some encouraging examples of consortium builds exist in LMICs, such as Ghana (Box 1).

**BOX 1 The future hospital: lessons from international practice.**

1 There are examples of successful hospital building programmes. Ghana is a LMIC in West Africa, with a population of 31 million. Following its first case of COVID‐19 in March 2020 there was a surge in patients needing hospitalisation, and the available treatment centres became overwhelmed. In response, Ghanaian business owners and corporate executives launched a campaign to construct a 100‐bed infectious disease hospital in less than 100 days, which would become Ghana's first modern health facility dedicated to infectious diseases. Architects from the Ghana Institute of Architects provided architectural services pro bono, the government provided the land next to a newly built district hospital in the capital, and the government provided tax waivers for various inputs. The Ghana COVID‐19 Private Sector Fund crowdsourced funds. The entire project cost US$7.5 million, with US$2 million being in‐kind contributions. The 100‐bed facility, including a 20‐bed Intensive Care Unit (ICU), laboratory and patient‐centred amenities, was constructed with locally sourced building materials and 80% of the contractors were Ghanaians. The design and construction incorporated sustainability features and the building is expected to use 28% less water and 23% less energy. The Ghana Infectious Disease Centre (GIDC) was completed in 91 days and was quickly operationalised with redeployed staff from other facilities as permanent staff were hired. The facility quickly became the primary location for the management of patients with moderate to severe COVID‐19 and it remains the largest Ghanian facility dedicated to infectious diseases. The GIDC demonstrates the potential for effective public‐private cooperation leveraging established strengths (private sector leading construction, public sector managing facility operations), political commitment and feasibility to pursue sustainable construction in LMICs. The GIDC is now under the management of the Ghana Health Service.

## 11 Staffing and Human Factors

Future hospital functionality goes beyond buildings and technology. While essential, a motivated, skilled workforce will only realise the benefits. A 2022 survey of over 2000 clinicians from 100 countries highlighted burnout and intent to leave [102]. Ageing populations and associated health needs will increase demand for healthcare services.

Future hospitals must prioritise recruitment and retention of staff by offering competitive terms and conditions [103]. In some countries, staff leave for sectors like technology, pharmaceuticals, and financial services, while others, especially in LMICs, are migrating to other countries' health systems. However, many countries also have large numbers of trained staff working informally or in the private sector due to poor conditions in the public sector [104]. This means providing competitive salaries, and in many countries where this does not happen, staff may extract additional funds through informal payments [105]. Yet while competitive salaries are important, they alone do not retain staff. Research has shown factors like career aspirations, working conditions, and local support drive decisions, especially in rural areas [79]. The concept of a ‘magnet hospital’ [106] where staff feel valued and hierarchies are flat has been linked to better patient outcomes [107, 108].In the UK, for example, low morale among doctors is often due to petty restrictions and unpredictable schedules [109]. Technology issues, such as electronic patient record systems, have also contributed to dissatisfaction, reducing morale and increasing burnout [110].

Staff spend the longest time in hospitals, and meaningful attempts to create person‐centred hospitals must account for their preferences as well as those of patients.

**FIGURE 1:**
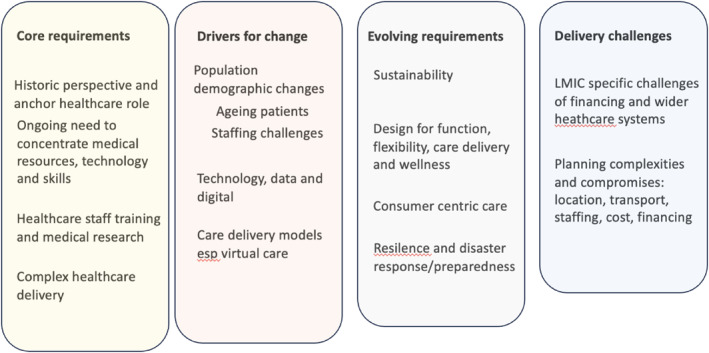
Framework for future hospital strategic decision making.

## 12 Recommendations for Policy

While the future is, by its nature, uncertain, we can make some broad recommendations that are forward‐looking and flexible. Future hospitals must be integrated into broader health systems, considering infrastructure, community, culture, staffing, and sustainability factors. Hospitals must prioritise patient needs and preferences as healthcare shifts towards a more patient‐centric model. Designing policies focussing on individual care enhances patient experience and outcomes, creating environments where patients feel valued and supported (Figure 1).

Sustainability and achieving net‐zero carbon goals should be central to future hospital planning. Policymakers must implement strategies to reduce hospitals' environmental impact, including energy‐efficient designs and sustainable resource management practices, which are essential for both high‐ and low‐income countries. If we are to avoid rural healthcare deserts, hospitals must be more accessible to rural populations, but this will require actions in employment policy and transport infrastructure. Technological advances, including telemedicine, must be embraced to enhance hospital design and operations. Policymakers should support integrating new technologies that improve healthcare delivery and patient outcomes, including investments in digital health. Above all, strategic planning is vital for resilient healthcare systems. This must include workforce planning, as recruitment, training, and retention strategies are crucial for maintaining a skilled workforce and ensuring high‐quality care. Policymakers should take a long‐term approach to avoid inequalities and ensure hospitals can respond effectively to future challenges and emergencies.

## 13 Conclusion

Future hospital development depends on leveraging complex infrastructures, considering the hospital's role in the local healthcare system and its interaction with other services. Building and estate infrastructure should incorporate evolving architectural concepts while prioritising sustainability in capital projects and ongoing costs. Digital technologies and novel healthcare delivery models, such as telemedicine and digital therapeutics, will influence design decisions, including IT infrastructure, environments, and skill requirements. Future hospital design must avoid widening health inequalities.

Hospitals will remain crucial in healthcare across countries at all levels of development. They cannot be viewed in isolation from broader health system changes, infrastructure, community and cultural factors, staffing, sustainability, and more. Hospitals impact healthcare planning, design, and delivery. We anticipate that future hospitals will enhance population health and support moves towards consumer‐centric healthcare. Clinical and policy planners should carefully consider these factors to maximise the potential of future hospitals to meet changing health needs.

## Author Contributions

N.J.S. and M.M. conceived the series and produced the first draft. All authors contributed to working groups, specialist content, draft development, and the writing of the final manuscript.

## Ethics Statement

Perspective hence ethical approval not required.

## Conflicts of Interest

The authors declare no conflicts of interest.

## Data Availability

The data that support the findings of this study are available on request from the corresponding author. The data are not publicly available due to privacy or ethical restrictions.

## References

[1] N. J. Sebire , A. Adams , L. Arpianen et al., “The Future Hospital in Global Health Systems: The Future Hospital as an Entity,” International Journal of Health Planning and Management (2025), 10.1002/hpm.3893. PMC12045724 39748156

[2] World Health Organization, Climate Change and Health (Geneva: World Health Organization), accessed December 30, 2024, https://www.who.int/teams/environment‐climate‐change‐and‐health/climate‐change‐and‐health/country‐support/climate‐resilient‐and‐environmentally‐sustainable‐health‐care‐facilities.

[3] R. Hutchings , S. Scobie , N. Edwards , Fit for the Future: What Can the NHS Learn about Digital Health Care from Other European Countries? (London: Nuffield Trust), accessed April 25, 2022, https://www.nuffieldtrust.org.uk/research/fit‐for‐the‐future‐what‐can‐the‐nhs‐learn‐about‐digital‐health‐care‐from‐other‐european‐countries.

[4] R. Moynihan , S. Sanders , Z. A. Michaleff , et al., “Impact of COVID‐19 Pandemic on Utilisation of Healthcare Services: A Systematic Review,” BMJ Open 11, no. 3 (2021): e045343, 10.1136/bmjopen-2020-045343. PMC7969768 33727273

[5] A. Sagan , S. Thomas , E. Webb , and M. McKee , “Assessing Resilience of a Health System Is Difficult but Necessary to Prepare for the Next Crisis,” BMJ 382 (2023): e073721, 10.1136/bmj-2022-073721. 37402509 PMC10316386

[6] Bangladesh Hospital Wins RIBA International Prize 2021 for the World’s Best New Building (London: RIBA), accessed December 30, 2024, https://www.architecture.com/knowledge‐and‐resources/knowledge‐landing‐page/bangladesh‐hospital‐wins‐riba‐international‐prize‐2021‐for‐the‐worlds‐best‐new‐building.

[7] World Health Organization, The Impact of COVID‐19 on Global Health Goals (Geneva: World Health Organization), accessed April 20, 2022, https://www.who.int/news‐room/spotlight/the‐impact‐of‐covid‐19‐on‐global‐health‐goals.

[8] World Health Organization, Rethinking the Future of Hospitals in the WHO European Region (Copenhagen: World Health Organization), accessed November 24, 2022, https://www.who.int/europe/news‐room/events/item/2022/04/21/default‐calendar/rethinking‐the‐future‐of‐hospitals‐in‐the‐who‐european‐region.

[9] The Future of Healthcare 2022 Infographic | HIMSS (Chicago, IL: HIMSS), accessed November 24, 2022, https://www.himss.org/resources/future‐healthcare‐2022‐infographic.

[10] Health Equipment – Hospital Beds –OECD Data (Paris: OECD), accessed August 2, 2022, https://data.oecd.org/healtheqt/hospital‐beds.htm.

[11] Pharmaccess, NIGERIA HEALTH SECTOR – Market Study Report, accessed December 30, 2024, https://www.pharmaccess.org/wp-content/uploads/2023/02/Nigeria-Healthcare-Market-Study-10-Case-StudiesProviders-link_Final-Report_2022.docx.pdf.

[12] World Health Organization, Indicator Metadata Registry Details (Geneva: World Health Organization), accessed May 16, 2023, https://www.who.int/data/gho/indicator‐metadata‐registry/imr‐details/3119.

[13] OECD , State of Health in the EU. Estonia Country Health Profile (Paris: OECD, 2017), https://eurohealthobservatory.who.int/docs/librariesprovider3/country‐health‐profiles/health‐profile‐estonia‐eng‐2017.pdf.

[14] F. Mubarak and R. Suomi , “Elderly Forgotten? Digital Exclusion in the Information Age and the Rising Grey Digital Divide,” Inquiry 59 (2022): 469580221096272, 10.1177/00469580221096272. 35471138 PMC9052810

[15] B. Rechel , S. Wright , J. Barlow , and M. McKee , “Hospital Capacity Planning: From Measuring Stocks to Modelling Flows,” Bulletin of the World Health Organization 88, no. 8 (2010): 632–636, 10.2471/blt.09.073361. 20680129 PMC2908974

[16] B. Rechel , S. Wright , N. Edwards , et al., Investing in Hospitals of the Future (Brussels: European Observatory on Health Systems and Policies), accessed July 25, 2023, https://eurohealthobservatory.who.int/publications/i/investing‐in‐hospitals‐of‐the‐future‐study.

[17] J. Manton and M. Gorsky , “Health Planning in 1960s Africa: International Health Organisations and the Post‐Colonial State,” Medical History 62, no. 4 (2018): 425–448, 10.1017/mdh.2018.41. 30191785 PMC6158634

[18] M. Powell , “Coasts and Coalfields: The Geographical Distribution of Doctors in England and Wales in the 1930s,” Social History of Medicine 18, no. 2 (2005): 245–263, 10.1093/sochis/hki032.

[19] J. Healy and M. McKee , eds. Accessing Health Care: Responding to Diversity. Illustrated edition (Oxford: OUP Oxford, 2004).

[20] Australian Institute of Health and Welfare , Chronic Health Conditions Among Culturally and Linguistically Diverse Australians, 2021 (Canberra: Australian Institute of Health and Welfare, 2023). accessed December 30, 2024, https://www.aihw.gov.au/reports/cald‐australians/chronic‐conditions‐cald‐2021/contents/about.

[21] J. Xu , M. Gorsky , and A. Mills , “A Path Dependence Analysis of Hospital Dominance in China (1949–2018): Lessons for Primary Care Strengthening,” Health Policy and Planning 35, no. 2 (2020): 167–179, 10.1093/heapol/czz145. 31778184

[22] M. E. Kruk , A. D. Gage , C. Arsenault , et al., “High‐quality Health Systems in the Sustainable Development Goals Era: Time for a Revolution,” Lancet Global Health 6, no. 11 (2018): e1196–e1252, 10.1016/s2214-109x(18)30386-3. 30196093 PMC7734391

[23] N. Edwards , Lessons from the Last Hospital Building Programme, and Recommendations for the Next (London: Nuffield Trust), accessed January 4, 2023, https://www.nuffieldtrust.org.uk/resource/lessons‐from‐the‐last‐hospital‐building‐programme‐and‐recommendations‐for‐the‐next.

[24] OECD . Health Statistics 2023 ‐ OECD (Paris: OECD), accessed August 30, 2023, https://www.oecd.org/health/health‐data.htm.

[25] M. T. Schneider , A. Y. Chang , A. Chapin , et al., “Health Expenditures by Services and Providers for 195 Countries, 2000–2017,” BMJ Global Health 6, no. 7 (2021): e005799, 10.1136/bmjgh-2021-005799. PMC8327839 34330760

[26] M. Giancotti , A. Guglielmo , and M. Mauro , “Efficiency and Optimal Size of Hospitals: Results of a Systematic Search,” PLoS One 12, no. 3 (2017): e0174533, 10.1371/journal.pone.0174533. 28355255 PMC5371367

[27] M. Gaynor . Examining the Impact of Health Care Consolidation’ Statement before the Committee on Energy and Commerce, Oversight and Investigations Subcommittee (U.S. House of Representatives, 2018), 10.2139/ssrn.3287848.

[28] M. Flojstrup , S. B. B. Bogh , M. Bech , D. P. Henriksen , S. P. Johnsen , and M. Brabrand , “Mortality Before and After Reconfiguration of the Danish Hospital‐Based Emergency Healthcare System: A Nationwide Interrupted Time Series Analysis,” BMJ Quality and Safety 32, no. 4 (2023): 202–213, 10.1136/bmjqs-2021-013881. PMC10086286 35589401

[29] L. Vaughan , M. Bardsley , D. Bell , et al., “Models of Generalist and Specialist Care in Smaller Hospitals in England: A Mixed‐Methods Study,” Health Services and Delivery Research 9, no. 4 (2021): 1–158, 10.3310/hsdr09040. 33651526

[30] E. A. Stewart , “On Loving the NHS,” in How Britain Loves the NHS (London: Policy Press, 2023), 1–15.

[31] E. Stewart , “A Sociology of Public Responses to Hospital Change and Closure,” Sociology of Health & Illness 41, no. 7 (2019): 1251–1269, 10.1111/1467-9566.12896. 30963595 PMC6849761

[32] M. J. Laugesen and R. Gauld, 2013. Democratic Governance and Health, accessed November 13, 2023, https://www.otago.ac.nz/press/books/democratic‐governance‐and‐health.

[33] MedPAC Chapter 15: Congressional Request on Health Care Provider Consolidation (March 2020 Report) (Washington, DC: Medpac, 2023), accessed August 30, 2023, https://www.medpac.gov/document/http‐www‐medpac‐gov‐docs‐default‐source‐reports‐mar20_medpac_ch15_sec‐pdf/.

[34] E. Nolte , E. Pitchforth , C. Miani , and S. McHugh , The Changing Hospital Landscape (Santa Monica, CA: RAND Corporation), accessed August 30, 2023, https://www.rand.org/pubs/periodicals/health‐quarterly/issues/v4/n3/01.html.

[35] T. Coker , Too Small to Play? M&A in the Nigerian HMO Space. TC HEALTH, accessed November 20, 2023, https://www.tchealthng.com/thought‐pieces/too‐small‐to‐play‐ma‐in‐the‐nigerian‐hmo‐space.

[36] M. Erasmus and N. Theron , “Market Concentration Trends in South Africa’s Private Healthcare Sector,” South African Journal of Economics and Management Sciences 19, no. 1 (2016): 53–63, 10.17159/2222-3436/2016/v19n1a4.

[37] E. Ferlie , “The New Public Management and Public Management Studies,” in Oxford Research Encyclopedia of Business and Management (Oxford: Oxford University Press, 2017), 10.1093/acrefore/9780190224851.013.129.

[38] A. Pilny , “Mergers and Acquisitions in the German Hospital Market – Who Are the Targets?,” SSRN Electronic Journal (2014), 10.2139/ssrn.2565703.

[39] A. Boutayeb , “The Burden of Communicable and Non‐communicable Diseases in Developing Countries,” Handbook of Disease Burdens and Quality of Life Measures (2010): 531–546, 10.1007/978-0-387-78665-0_32.

[40] World Bank , Universal Health Coverage (Washington, DC: World Bank), accessed June 16, 2023, https://www.worldbank.org/en/topic/universalhealthcoverage.

[41] P. O. Otieno , G. Asiki , P. O. Otieno , and G. Asiki , “Making Universal Health Coverage Effective in Low‐ and Middle‐Income Countries: A Blueprint for Health Sector Reforms,” in Healthcare Access ‐ Regional Overviews (London: IntechOpen, 2020), 10.5772/intechopen.91414.

[42] Project Access – Global Surgery Research (Birmingham, UK: NIHR Global Health Research Unit on Global Surgery), accessed June 16, 2023, https://www.globalsurgeryunit.org/clinical‐trials‐holding‐page/project‐access/.

[43] J. G. Meara , A. J. M. Leather , L. Hagander , et al., “Global Surgery 2030: Evidence and Solutions for Achieving Health, Welfare, and Economic Development,” Lancet 386, no. 9993 (2015): 569–624, 10.1016/s0140-6736(15)60160-x. 25924834

[44] C. E. Grimes , K. G. Bowman , C. M. Dodgion , and C. B. D. Lavy , “Systematic Review of Barriers to Surgical Care in Low‐Income and Middle‐Income Countries,” World Journal of Surgery 35, no. 5 (2011): 941–950, 10.1007/s00268-011-1010-1. 21360305

[45] World Health Organization, Monitoring Emergency Obstetric Care: A Handbook (Geneva: World Health Organization), accessed June 16, 2023, https://www.who.int/publications‐detail‐redirect/9789241547734.

[46] K. Murugiah , S. V. Nuti , and H. M. Krumholz , “STEMI Care in LMIC – Obstacles and Opportunities,” Glob Heart 9, no. 4 (2014): 429–430, 10.1016/j.gheart.2014.08.010. 25592797 PMC5459383

[47] T. Alexander , A. Mullasari , and B. Nallamothu , “Management Strategies for Acute STEMI in Low‐ and Middle‐Income Countries: Experience of the Tamil Nadu ST‐Segment Elevation Myocardial Infarction Programme,” AsiaIntervention 7, no. 1 (2021): 27–34, 10.4244/aij-d-21-00008. 34912999 PMC8670567

[48] R. K. Kayambankadzanja , C. O. Schell , I. Mbingwani , S. K. Mndolo , M. Castegren , and T. Baker , “Unmet Need of Essential Treatments for Critical Illness in Malawi,” PLoS One 16, no. 9 (2021): e0256361, 10.1371/journal.pone.0256361. 34506504 PMC8432792

[49] C. Aluttis , T. Bishaw , and M. W. Frank , “The Workforce for Health in a Globalized Context – Global Shortages and International Migration,” Global Health Action 7, no. 1 (2014), 10.3402/GHA.V7.23611. PMC3926986 24560265

[50] World Health Organization , Global Strategy on Human Resources for Health: Workforce 2030 (Geneva: World Health Organization, 2016), https://apps.who.int/iris/handle/10665/250368.

[51] C. Meadows , W. Rattenberry , and C. Waldmann , “Centralisation of Specialist Critical Care Services,” Journal of the Intensive Care Society 12, no. 2 (2011): 87–89, 10.1177/175114371101200202.

[52] A. Banke‐Thomas , K. Wright , O. Sonoiki , et al., “Assessing Emergency Obstetric Care Provision in Low‐ and Middle‐Income Countries: A Systematic Review of the Application of Global Guidelines,” Global Health Action 9, no. 1 (2016): 31880, 10.3402/gha.v9.31880. 27498964 PMC4976306

[53] I. J. Mungall , “Trend towards Centralisation of Hospital Services, and its Effect on Access to Care for Rural and Remote Communities in the UK,” Rural and Remote Health 5 (2005): 390. 15913450

[54] D. Modonutti , V. M. Ramakrishnan , and Q.‐D. Trinh , “All for One, One for All: Is Centralisation the Way to Go?,” BJU International 125 (2020): 191–192, 10.1111/bju.14973. 31943684

[55] A. Alakija , “Leveraging Lessons From the COVID‐19 Pandemic to Strengthen Low‐Income and Middle‐Income Country Preparedness for Future Global Health Threats,” Lancet Infectious Diseases 23, no. 8 (2023): e310–e317, 10.1016/S1473-3099(23)00279-7. 37290474

[56] J. Langenbrunner , C. Cashin , and S. O’Dougherty , Designing and Implementing Health Care Provider Payment Systems How ‐To Manuals (Washington, DC: World Bank, 2009), https://documents1.worldbank.org/curated/en/577441468169753880/pdf/543180BRI0Box31ymentMethodsoverview.pdf.

[57] R. Busse , A. Geissler , A. Aaviksoo , et al., “Diagnosis Related Groups in Europe: Moving towards Transparency, Efficiency, and Quality in Hospitals?,” BMJ 346, no. jun07 3 (2013): f3197, 10.1136/bmj.f3197. 23747967

[58] Marshall L. , Charlesworth A. , Hurst J. , “The NHS Payment System: Evolving Policy and Emerging Evidence,”.

[59] Shortell S. , Addicott R. , Walsh N. , Ham C. , “Accountable Care Organisations in the United States and England,”.

[60] D. Campbell , ed. DCH Policy. Johnson’s ‘40 New Hospitals’ Pledge Costed at up to £24bn (London: Guardian, 2019), https://www.theguardian.com/politics/2019/dec/08/boris‐johnson‐40‐new‐hospitals‐pledge‐costed.

[61] Agenda 111 Hospitals: Each Hospital to Cost US$16.88million — Akufo‐Addo. Modern Ghana (Accra: Modern Ghana), accessed August 8, 2023, https://www.modernghana.com/news/1098717/agenda‐111‐hospitals‐each‐hospital‐cost‐us1688m.html.

[62] J. Zheng , J. Roehrich , and M. Lewis , The Dynamics of Contractual and Relational Governance: Evidence from Long‐Term Public‐Private Procurement Arrangements (Rochester, NY: SSRN, 2008), https://papers.ssrn.com/abstract=2951835.

[63] M. McKee , N. Edwards , and R. Atun , “Public‐private Partnerships for Hospitals,” Bulletin of the World Health Organization 84 (2006): 890–896. 17143463 PMC2627548

[64] R. A. Atun and M. McKee , “Is the Private Finance Initiative Dead?,” BMJ 331, no. 7520 (2005): 792–793, 10.1136/bmj.331.7520.792. 16210259 PMC1246066

[65] M. Goodier , NHS Hospital Trusts Paying Hundreds of Millions in Interest to Private Firms (London: Guardian, 2022), https://www.theguardian.com/politics/2022/oct/25/nhs‐hospital‐trusts‐paying‐hundreds‐of‐millions‐in‐interest‐to‐private‐firms.

[66] M. Wilson , A. Guta , K. Waddell , J. Lavis , R. Reid , and C. Evans , “The Impacts of Accountable Care Organizations on Patient Experience, Health Outcomes and Costs: A Rapid Review,” Journal of Health Services Research and Policy 25, no. 2 (2020): 130–138, 10.1177/1355819620913141. 32321282

[67] C. Mora , T. McKenzie , I. M. Gaw , et al., “Over Half of Known Human Pathogenic Diseases Can Be Aggravated by Climate Change,” Nature Climate Change 12, no. 9 (2022): 869–875, 10.1038/s41558-022-01426-1. PMC9362357 35968032

[68] M. Lenzen , A. Malik , M. Li , et al., “The Environmental Footprint of Health Care: A Global Assessment,” Lancet Planetary Health 4, no. 7 (2020): e271–e279, 10.1016/s2542-5196(20)30121-2. 32681898

[69] J. Jubb , The Nexus Between Climate Change and Healthcare (Health Policy Partnership, 2022), https://www.healthpolicypartnership.com/the‐nexus‐between‐climate‐change‐and‐healthcare/.

[70] NHS English , Delivering a Net Zero NHS (London: NHS England), accessed January 3, 2023, https://www.england.nhs.uk/greenernhs/a‐net‐zero‐nhs/.

[71] COP26 Health Programme, accessed June 6, 2023, https://www.who.int/initiatives/alliance‐for‐transformative‐action‐on‐climate‐and‐health/cop26‐health‐programme.

[72] A. García‐Altés , M. McKee , L. Siciliani , et al., “Understanding Public Procurement Within the Health Sector: A Priority in a Post‐COVID‐19 World,” Health Economics, Policy and Law 18, no. 2 (2023): 172–185, 10.1017/s1744133122000184. 35894208

[73] H. Rahimi‐Ardabili , F. Magrabi , and E. Coiera , “Digital Health for Climate Change Mitigation and Response: A Scoping Review,” Journal of the American Medical Informatics Association 29, no. 12 (2022): 2140–2152, 10.1093/jamia/ocac134. 35960171 PMC9667157

[74] Z. Ullah , A. R. Nasir , F. K. Alqahtani , F. Ullah , M. J. Thaheem , and A. Maqsoom , “Life Cycle Sustainability Assessment of Healthcare Buildings: A Policy Framework,” Buildings 13, no. 9 (2023): 2143, 10.3390/buildings13092143.

[75] Y. H. Yau , D. Chandrasegaran , and A. Badarudin , “The Ventilation of Multiple‐Bed Hospital Wards in the Tropics: A Review,” Building and Environment 46, no. 5 (2011): 1125–1132, 10.1016/j.buildenv.2010.11.013. 32288016 PMC7116949

[76] F. Ibrahim , E. Z. Samsudin , A. R. Ishak , and J. Sathasivam , “Hospital Indoor Air Quality and its Relationships With Building Design, Building Operation, and Occupant‐Related Factors: A Mini‐Review,” Frontiers in Public Health 10 (2022): 1067764, 10.3389/fpubh.2022.1067764. 36424957 PMC9679624

[77] E. Coiera and J. Braithwaite , “Turbulence Health Systems: Engineering a Rapidly Adaptive Health System for Times of Crisis,” BMJ Health Care Inform 28, no. 1 (2021): e100363, 10.1136/bmjhci-2021-100363. PMC8382666 34417204

[78] B. Rechel , J. Buchan , and M. McKee , “The Impact of Health Facilities on Healthcare Workers’ Well‐Being and Performance,” International Journal of Nursing Studies 46, no. 7 (2009): 1025–1034, 10.1016/j.ijnurstu.2008.12.008. 19166997

[79] B. Angell , M. Khan , R. Islam , et al., “Incentivising Doctor Attendance in Rural Bangladesh: A Latent Class Analysis of a Discrete Choice Experiment,” BMJ Global Health 6, no. 7 (2021): e006001, 10.1136/bmjgh-2021-006001. PMC8323362 34326070

[80] World Health Organization , Health Workforce (Geneva: World Health Organization), accessed May 17, 2023, https://www.who.int/health‐topics/health‐workforce.

[81] T. Brooking , The History of New Zealand (Bloomsbury) (London: Bloomsbury), accessed August 8, 2023, https://www.bloomsbury.com/uk/history‐of‐new‐zealand‐9780313058493/.

[82] E. Hanson . “The Politics of Social Security: The 1938 Act and Some Later Developments,”. National Library of New Zealand, accessed November 13, 2023, https://natlib.govt.nz/records/21103753.

[83] G. Rivett , The History of the NHS (London: Nuffield Trust), accessed May 17, 2023, https://www.nuffieldtrust.org.uk/health‐and‐social‐care‐explained/the‐history‐of‐the‐nhs.

[84] N. Klein , The Shock Doctrine: The Rise of Disaster Capitalism. 1st ed. (London: Penguin, 2008).

[85] N. Edwards and R. B. Saltman , “Re‐thinking Barriers to Organizational Change in Public Hospitals,” Israel Journal of Health Policy Research 6, no. 1 (2017): 8, 10.1186/s13584-017-0133-8. 28321291 PMC5357814

[86] A. S. Preker , A. Harding , and P. Travis , “Make or Buy’ Decisions in the Production of Health Care Goods and Services: New Insights From Institutional Economics and Organizational Theory,” Bulletin of the World Health Organization 78 (2000): 779–790. 10916915 PMC2560779

[87] O. E. Williamson , “Opportunism and its Critics,” Managerial and Decision Economics 14, no. 2 (1993): 97–107, 10.1002/mde.4090140203.

[88] K. J. Arrow , “Uncertainty and the Welfare Economics of Medical Care,” American Economic Review 53 (1963): 941–973.

[89] D. W. Simborg , “DRG Creep: A New Hospital‐Acquired Disease,” New England Journal of Medicine 304, no. 26 (1981): 1602–1604, 10.1056/nejm198106253042611. 7015136

[90] M. McKee and D. Stuckler , “The Assault on Universalism: How to Destroy the Welfare State,” BMJ 343, (2011): d7973, 10.1136/bmj.d7973. 22187190

[91] A. Reeves , M. McKee , and D. Stuckler , “The Attack on Universal Health Coverage in Europe: Recession, Austerity and Unmet Needs,” European Journal of Public Health 25, no. 3 (2015): 364–365, 10.1093/eurpub/ckv040. 25999461 PMC4440451

[92] H. Legido‐Quigley , N. Pocock , S. T. Tan , et al., “Healthcare Is Not Universal if Undocumented Migrants Are Excluded,” BMJ 366 (2019): l4160, 10.1136/bmj.l4160. 31527060 PMC6741752

[93] E. Okiro and P. Ouma , People across Africa Have to Travel Far to Get to a Hospital. We Worked Out How Far (London: Conversation, 2018), http://theconversation.com/people‐across‐africa‐have‐to‐travel‐far‐to‐get‐to‐a‐hospital‐we‐worked‐out‐how‐far‐102585.

[94] T. A. H. Rocha , N. C. da Silva , P. V. Amaral , et al., “Addressing Geographic Access Barriers to Emergency Care Services: A National Ecologic Study of Hospitals in Brazil,” International Journal for Equity in Health 16, no. 1 (2017): 149, 10.1186/s12939-017-0645-4. 28830521 PMC5568346

[95] S. Chawla , S. Kurani , S. M. Wren , et al., “Electricity and Generator Availability in LMIC Hospitals: Improving Access to Safe Surgery,” Journal of Surgical Research 223 (2018): 136–141, 10.1016/j.jss.2017.10.016. 29433865 PMC13064570

[96] S. T. Syed , B. S. Gerber , and L. K. Sharp , “Traveling towards Disease: Transportation Barriers to Health Care Access,” Journal of Community Health 38, no. 5 (2013): 976–993, 10.1007/s10900-013-9681-1. 23543372 PMC4265215

[97] H. Alami , L. Rivard , P. Lehoux , et al., “Artificial Intelligence in Health Care: Laying the Foundation for Responsible, Sustainable, and Inclusive Innovation in Low‐ and Middle‐Income Countries,” Globalization and Health 16, no. 1 (2020): 52, 10.1186/s12992-020-00584-1. 32580741 PMC7315549

[98] N. Schwalbe and B. Wahl , “Artificial Intelligence and the Future of Global Health,” Lancet 395, no. 10236 (2020): 1579–1586, 10.1016/s0140-6736(20)30226-9. 32416782 PMC7255280

[99] D. M. López , C. Rico‐Olarte , B. Blobel , and C. Hullin , “Challenges and Solutions for Transforming Health Ecosystems in Low‐ and Middle‐Income Countries through Artificial Intelligence,” Frontiers of Medicine 9 (2022): 958097, 10.3389/fmed.2022.958097. PMC9755337 36530888

[100] A. Farlow , A. Hoffmann , G. A. Tadesse , et al., “Rethinking Global Digital Health and AI‐For‐Health Innovation Challenges,” PLOS Global Public Health 3, no. 4 (2023): e0001844, 10.1371/journal.pgph.0001844. 37115743 PMC10146484

[101] Boseley S. , ed. health. Half of Lesotho Health Budget Goes to Private Consortium for One Hospital (London: Guardian, 2014), https://www.theguardian.com/world/2014/apr/07/lesotho‐health‐budget‐private‐consortium‐hospital.

[102] Elsevier Health, Clinician of the Future: A 2022 Report (Amsterdam: Elsevier), accessed April 26, 2022, https://www.elsevier.com/connect/clinician‐of‐the‐future.

[103] T. Zapata , N. Azzopardi‐Muscat , M. McKee , and H. Kluge , “Fixing the Health Workforce Crisis in Europe: Retention Must Be the Priority,” BMJ 381 (2023): 947, 10.1136/bmj.p947. 37185627

[104] E. Hutchinson , S. Kiwanuka , R. Muhindo , et al., “The Paradoxical Surplus of Health Workers in Africa: The Need for Research and Policy Engagement,” International Journal of Health Planning and Management 39, no. 3 (2024): 956–962, 10.1002/hpm.3745. 38193753

[105] P. Gaal and M. McKee , “Informal Payment for Health Care and the Theory of ‘INXIT’,” International Journal of Health Planning and Management 19, no. 2 (2004): 163–178, 10.1002/hpm.751. 15239210

[106] J. G. Scott , J. Sochalski , and L. Aiken , “Review of Magnet Hospital Research: Findings and Implications for Professional Nursing Practice,” Journal of Nursing Administration 29, no. 1 (1999): 9–19, 10.1097/00005110-199901000-00003. 9921144

[107] M. D. McHugh , L. A. Kelly , H. L. Smith , E. S. Wu , J. M. Vanak , and L. H. Aiken , “Lower Mortality in Magnet Hospitals,” Medical Care 51, no. 5 (2013): 382–388, 10.1097/mlr.0b013e3182726cc5. 23047129 PMC3568449

[108] A. W. Stimpfel , D. M. Sloane , M. D. McHugh , and L. H. Aiken , “Hospitals Known for Nursing Excellence Associated With Better Hospital Experience for Patients,” Health Services Research 51, no. 3 (2016): 1120–1134, 10.1111/1475-6773.12357. 26369862 PMC4874824

[109] J. Spiers , F. Kokab , M. Buszewicz , et al., “Recommendations for Improving the Working Conditions and Cultures of Distressed Junior Doctors, Based on a Qualitative Study and Stakeholder Perspectives,” BMC Health Services Research 22, no. 1 (2022): 1333, 10.1186/s12913-022-08728-2. 36357890 PMC9647238

[110] R. L. Gardner , E. Cooper , J. Haskell , et al., “Physician Stress and Burnout: The Impact of Health Information Technology,” Journal of the American Medical Informatics Association 26, no. 2 (2018): 106–114, 10.1093/jamia/ocy145. PMC7647171 30517663

